# Physicochemical and Microbiological Properties of Yoghurt Made Form Defatted Milk Mixtures and Concentrated Buttermilk Fortified with Herbal Extract

**DOI:** 10.3390/foods15162768

**Published:** 2026-08-07

**Authors:** Tamara Tultabayeva, Gulmira Zhakupova, Assem Sagandyk, Kadyrzhan Makangali, Alfiya Syzdykova, Aisarat Gajimuradova, Aknur Muldasheva, Kalamkas Dairova

**Affiliations:** 1 International Center, University of California, Davis, CA 95616, USA; 2 Institute of Engineering and Food Technology, Saken Seifullin Kazakh Agrotechnical University, Astana 010000, Kazakhstan

**Keywords:** functional yoghurt, *Plantago major* L., *Inula helenium* L., *Melissa officinalis* L., buttermilk concentrate, lactic acid bacteria viability, antimicrobial activity, prebiotic effect

## Abstract

This study evaluates the impact of plant-derived additives (*Melissa officinalis* L., *Plantago major* L., and *Inula helenium* L.) on the physicochemical, microbiological, and antimicrobial properties of yoghurt formulated with a combined base of milk and buttermilk concentrate (solid content 8–10%). Over 14 days of refrigerated storage, all fortified yoghurts maintained pH within acceptable limits, while *Plantago* and *Inula* slightly enhanced post-acidification without compromising quality. Notably, the buttermilk concentrate significantly boosted initial lactic acid bacteria (LAB) viability, and the yoghurt with *Plantago major* L. extract showed a 314% higher viable LAB count during storage compared with the control. In contrast, *Inula helenium* L. reduced LAB viability by 67.6%, indicating a trade-off between bioactivity and starter compatibility. Antimicrobial testing via disk-diffusion revealed selective activity against Gram-positive *Staphylococcus aureus* (maximum inhibition radius: 5.8 ± 0.5 mm for *Plantago* yoghurt), with no effect on Gram-negative *E. coli* or *P. aeruginosa*. Activity was driven primarily by acid-dependent metabolites, with *Plantago* retaining residual non-acidic bioactivity after neutralization. The combination of *Plantago major* L. with buttermilk concentrate emerged as optimal for functional yoghurt, enhancing higher viability of lactic acid bacteria during refrigerated storage. These findings support the use of compatible plant–dairy combinations to develop yoghurt products with improved physicochemical properties, LAB viability and microbiological stability under the tested conditions.

## 1. Introduction

Protein-enriched yoghurts are increasingly demanded by health-conscious consumers seeking convenient products with improved nutritional and functional quality, particularly higher protein content and reduced need for additives [1,2,3]. High-milk-protein preparations are widely used to increase total solids, enhance gel firmness, viscosity, and water-holding capacity, and to reduce syneresis in yoghurt while maintaining a relatively low fat content and a clean-label profile [3,4]. At the same time, there is growing interest in valorising dairy by-products as alternative protein sources for functional foods, addressing both economic and environmental pressures in the dairy sector [5,6].

Buttermilk, the serum phase obtained during butter manufacture, is a promising yet underutilized by-product. It is rich in milk proteins and residual lipids, including milk fat globule membrane components, and therefore possesses valuable techno-functional and nutritional properties [7,8]. However, due to its high organic load and seasonal or plant-specific variability, buttermilk is often treated as effluent, contributing to the overall pollution potential of dairy wastewater streams and increasing disposal costs [9]. Converting buttermilk into concentrated ingredients suitable for fortification of fermented dairy products represents a sustainable strategy for by-product valorisation, potentially improving protein content, texture, and stability of yoghurt while reducing environmental impact [3,5,10].

In parallel, the incorporation of plant-derived extracts into dairy matrices has gained significant attention as an approach to develop functional yoghurts with added health benefits. Herbal and fruit extracts are rich in phenolic compounds and other bioactive constituents that can contribute antioxidant and, in many cases, antimicrobial activities [11,12]. Previous studies have demonstrated that the addition of plant extracts to yoghurt can substantially increase total phenolic content and DPPH radical-scavenging activity, while maintaining or even improving microbiological stability during refrigerated storage [13,14]. Depending on the plant species and concentration, these extracts may also modulate color, flavor, and overall sensory acceptance, which are critical for consumer uptake of functional products [15].

In this study, we focused on three medicinal plants that are widely distributed in Northern Kazakhstan and traditionally used in local herbal preparations: *Melissa officinalis* L., *Plantago major* L. and *Inula helenium* L. These species were selected because their extracts are known to be rich in phenolic compounds and non-starch polysaccharides (e.g., mucilaginous fibres and inulin-type fructans), which may influence yoghurt structure, water retention and the viability of lactic acid bacteria, while their incorporation into fermented dairy products has been only sporadically investigated, leaving a knowledge gap regarding their behaviour in a yoghurt matrix. Combining high-protein fortification with dairy by-products and herbal extracts in a single yoghurt system is particularly attractive from both technological and nutritional perspectives: a buttermilk-based protein concentrate may enhance gel network formation and water-holding capacity, leading to improved firmness and reduced syneresis, whereas herbal extracts may provide additional antioxidant protection and contribute to the microbiological stability of the product [3,5,14]. Nevertheless, interactions between milk proteins, phenolic compounds and starter cultures are complex and can affect acidification kinetics, viability of lactic acid bacteria and the stability of bioactive compounds during storage [13,14,15,16,17], and systematic data on yoghurt simultaneously enriched with buttermilk concentrate and herbal extracts remain limited, particularly regarding the combined effects on structure, microbiological quality and antioxidant-related properties.

However, most previous studies have focused either on the use of buttermilk or buttermilk powder as a functional ingredient in conventional or low-fat yoghurts, or on the incorporation of individual plant extracts into standard milk bases, considering these approaches separately. Systematic data on high-protein yoghurts simultaneously enriched with a buttermilk-derived protein concentrate and herbal extracts are still scarce, particularly with regard to the combined effects of these ingredients on gel structure, water-holding capacity, water activity, and microbiological quality during refrigerated storage. Therefore, this study aims to address this gap by evaluating the physicochemical and microbiological behaviour of yoghurt formulated with a concentrated milk–buttermilk base and three selected herbal extracts during 10 days of refrigerated storage.

Therefore, this study aimed to develop yoghurt formulated with a concentrated milk–buttermilk base and selected herbal extracts and to evaluate how this combination affects the physicochemical properties, starter culture viability and antimicrobial activity of the product during refrigerated storage. Building on the literature, we hypothesized that the buttermilk-derived protein concentrate would strengthen the yoghurt gel network and reduce syneresis by supplying additional milk proteins, whereas the herbal extracts, as sources of phenolic compounds and non-starch polysaccharides, could modulate redox-related properties and influence the survival of lactic acid bacteria, provided that their interactions with the starter culture remain compatible.

## 2. Materials and Methods

### 2.1. Materials

Skimmed cow’s milk and skimmed goat’s milk were obtained from a local dairy plant in (Astana, Kazakhstan). A sweet-cream buttermilk concentrate was produced from pasteurised cream buttermilk after churning and separation, without additional defatting. The direct-to-vessel starter culture (*Streptococcus thermophilus*, *Lactobacillus delbrueckii* subsp. *bulgaricus*) (MicroMilk Yo Probi, 50U, Cremosano, Italy) and probiotic culture *L. reuteri* (Ivan Pole, 2 × 10^8^ CFU/per stick, Krasnogorsk, Russia) was stored and reactivated according to the supplier’s instructions.

Dried leaves and leafy stems of lemon balm (*Melissa officinalis* L.), dried leaves of plantain (*Plantago major* L.) and dried rhizomes with roots of elecampane (*Inula helenium* L.) were purchased as a herbal tea from a local pharmacy (Astana, Kazakhstan).

### 2.2. Sample Preparation

To develop a fermented milk product with an increased protein content, a multicomponent base consisting of skimmed cow’s milk, goat’s milk and buttermilk concentrate in a ratio of 50:20:30 was used. The ratio 50:20:30 (skimmed cow’s milk: skimmed goat’s milk: buttermilk concentrate) was selected based on optimisation experiments conducted within the framework of the research project, in which different blends were screened for gel formation, syneresis and sensory acceptability, and this composition provided the best balance between high solids, buttermilk enrichment and technological performance.

Buttermilk concentrate was prepared from fresh sweet-cream buttermilk obtained immediately after churning pasteurized cream in the university pilot dairy plant. Fresh buttermilk was concentrated by ultrafiltration using a tangential-flow system Vivaflow 50R (Sartorius, Göttingen, Germany) equipped with a 300 kDa membrane until the total solids content reached 8–10%. This cut-off was selected based on preliminary optimisation experiments conducted within the research project: smaller pore sizes led to rapid fouling and blockage of the membrane by buttermilk proteins, which severely reduced permeate flux, whereas larger pore sizes substantially increased the time needed to reach the target total solids content of 8–10% in the retentate. The 300 kDa membrane thus represented a compromise between protein retention efficiency, flux stability and processing time. Ultrafiltration was carried out at 35 °C in membrane modules according to the manufacturer’s recommendations. The resulting buttermilk concentrate was stored at 4 °C under refrigeration until use (not longer than 3 days), under the same conditions for all batches. Chemical composition of initial raw materials for combined blend of milk shown in Table 1.

A direct-vat set yoghurt starter culture and *L. reuteri* probiotic culture was used for inoculation in accordance with the manufacturer’s instructions. Fermentation was carried out at 40–42 °C in a thermostatic chamber TS-150 (Chelyabinsk Laboratory Equipment Plant, Chelyabinsk, Russia) until the pH reached 4.6–4.8.

Melissa (*Melissa officinalis* L.), Plantain (*Plantago major* L.) and Elecampane (*Ínula helénium* L.) extract was obtained by maceration in 40% (*v*/*v*) ethanol at a plant-to-solvent ratio of 1:10 (*w*/*v*). The mixture was kept for 24 h at room temperature under occasional stirring and then filtered through Whatman No. 1 filter paper. The filtrate was concentrated under reduced pressure using a rotary vacuum evaporator (Rotavapor R-300, Büchi, Flawil, Switzerland). Extracts were evaporated under reduced pressure and dried at 35 °C until the concentrate reached approximately 8–10% dry matter, and the yield was calculated as g of dried extract per 100 g of dried plant material to standardise the dosage across batches. Then the extract was stored at 4 °C in a sealed container until further use. Under these conditions, the hydroalcoholic solvent is effectively removed; no ethanol smell was detected in the dried extracts or in the fortified yoghurts, and LAB growth data did not indicate any inhibitory effect attributable to residual ethanol.

**Figure 1:**
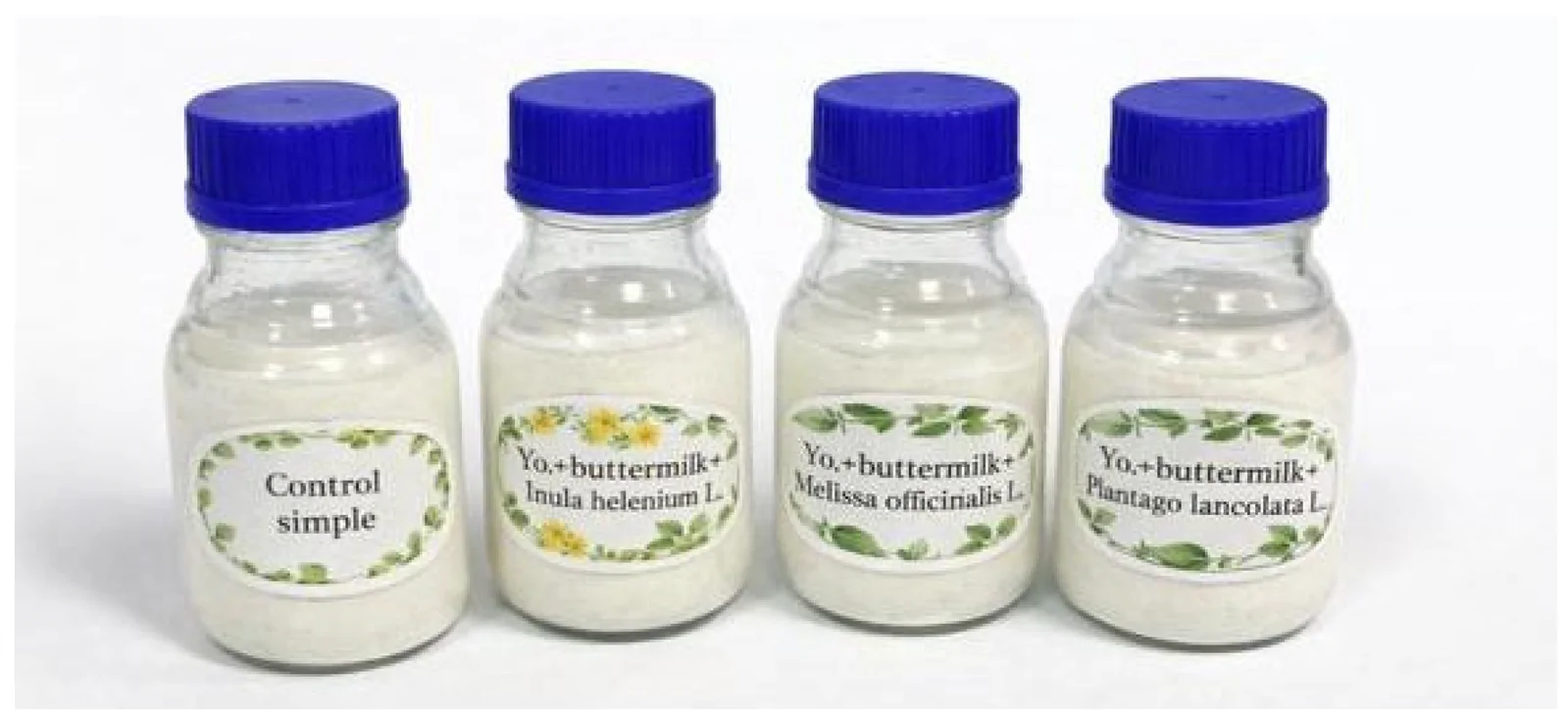
Pictures of resulting yoghurt.

The herbal extracts were added to the milk–buttermilk base at a level of 0.4% (*w*/*w*) immediately before inoculation, and the mixture was gently stirred for 2–3 min to ensure homogeneous dispersion of the extracts. After inoculation with the starter culture and L. Reuteri,, the yoghurt mix was incubated under static conditions until the desired pH was reached, without further stirring after gel formation, in order to avoid mechanical disruption of the coagulum and excessive whey separation. In this study, control yoghurt (YC) from a combined mixture of milk and buttermilk concentrate, yoghurt with Melissa extract from a combined mixture of milk and buttermilk concentrate (YM), yoghurt with plantain extract from a combined mixture of milk and buttermilk concentrate (YP), and yoghurt with elecampane extract from a combined mixture of milk and buttermilk concentrate (YE) were used. The resulting yoghurts are shown in Figure 1.

### 2.3. Determination of Nutrition Profile

Protein, fat, ash, moisture and carbohydrate contents were measured by FT-NIR spectroscopy (Bruker Tango, Bruker, Karlsruhe, Germany) using the manufacturer’s MilkPowder calibration models; the FTIR carbohydrate value (predominantly lactose and other soluble carbohydrates) was used as the carbohydrate fraction for subsequent energy calculations. Total energy was then calculated using the Atwater general factors according to the equation: energy (kcal/100 g yogurtt) = 4 × (g protein + g carbohydrates) + 9 × (g fat), in line with FAO/WHO recommendations for food composition and energy conversion [18].

### 2.4. Determination of Active and Titratable Acidity

Active acidity (pH) and titratable acidity (TA) of yoghurt samples were determined according to standard acid–base titration procedures commonly used for fermented milks, with minor modifications. Active acidity was measured using a digital pH meter (Seven2Go, Mettler Toledo, Greifensee, Switzerland). The instrument was calibrated daily with standard buffer solutions at pH 4.0 and 7.0 at room temperature. Yoghurt samples were gently stirred to obtain a homogeneous mass, and the pH was recorded by immersing the electrode directly into the sample and allowing the reading to stabilize.

Titratable acidity was determined by titration and expressed as g lactic acid per 100 g of yoghurt. An accurately weighed amount of yoghurt (10 g) was transferred into a beaker and diluted with 90 mL of distilled water at room temperature. The suspension was gently mixed and titrated with standardized 0.1 N NaOH solution under continuous stirring, using phenolphthalein as an indicator, until a persistent faint pink colour was observed for at least 30 s (corresponding to an endpoint of approximately pH 8.3). Titratable acidity was calculated using the following equation:

(1) $$\mathrm{TA}\;(\%\;\mathrm{lactic}\;\mathrm{acid})=\frac{VNaOH\times NNaOH\times 0.09}{msample}\times 100,$$

where V_NaOH_ is the volume of NaOH used for titration (mL),

N_NaOH_ is the normality of the NaOH solution,

0.09 is the equivalent weight of lactic acid (g/mmol),

m_sample_ is the mass of the yoghurt sample (g) [19].

### 2.5. Determination of Water-Holding Capacity (WHC)

The water-holding capacity (WHC) of yoghurt samples was determined using a centrifugation method adapted from Sodini et al. [20]. Before analysis, yoghurt samples were gently stirred to ensure homogeneity, avoiding excessive disruption of the gel structure. An accurately weighed portion of yoghurt (e.g., 20.0 ± 0.1 g) was transferred into a centrifuge tube and centrifuged in centrifuge 5810 R (Eppendorf, Hamburg, Germany) at 1250× *g* for 10 min at 4 °C. After centrifugation, the expelled whey was carefully decanted and weighed. WHC was calculated according to the following equation and expressed as percentage:

(2) $$\mathrm{WHC}\;(\%)=100\times \frac{myoghurt-mwhey}{myoghurt},$$

where m_yoghurt_ is the initial mass of the yoghurt sample (g),

m_whey_ is the mass of separated whey (g).

### 2.6. Determination of Syneresis

Syneresis of yoghurt samples was determined using a centrifugal method, as described previously with minor modifications. Briefly, an accurately weighed amount of yoghurt (e.g., 20.0 ± 0.1 g) was transferred into a 50 mL centrifuge tube, taking care to minimize disruption of the gel structure. The samples were centrifuged (5810 R, Eppendorf, Germany) at 4000× *g* for 15 min at 10 °C. After centrifugation, the separated whey was carefully decanted and weighed. Syneresis was expressed as the percentage of whey expelled relative to the initial mass of yoghurt, according to the following equation:

(3) $$\mathrm{Syneresis}\;(\%)=100\times \frac{mwhey}{myogurt}$$

where m_whey_ is the mass of separated whey (g),

m_yoghurt_ is the mass of the yoghurt sample [21].

### 2.7. Determination of Water Activity (aw)

Water activity (*a_w_*) of yoghurt enriched with plant extract was determined using an Aqualab 4TE water activity meter (Meter Group, Inc., Pullman, Washington, WA, USA) at 25 °C. Prior to measurement, samples were equilibrated in the instrument chamber for 15 min, and a_w_ values were recorded directly from the display; all measurements were performed in triplicate in accordance with the manufacturer’s instructions. Water activity was evaluated as an indicator of microbiological stability during refrigerated storage, since lower a_w_ values are generally associated with a reduced growth potential of spoilage microorganisms in fermented dairy products [22].

### 2.8. Determination of Colony-Forming Units (CFU)

The number of viable lactic acid bacteria was determined by the surface-plating method on MRS agar Sigma-Aldrich (Merck Group, Rahway, NJ, USA). Serial 10-fold dilutions of the yoghurt samples were prepared in sterile peptone water, and 0.1 mL of each dilution was spread onto MRS agar plates. Plates were incubated at 37 °C for 48 h under aerobic conditions, and colonies were then counted. Plates containing 30–300 colonies were selected for calculations; when two consecutive dilutions met this criterion, the CFU/mL value was calculated using the mean colony count. The number of colony-forming units per mL of sample was calculated according to ISO 4833-1:

(4) $$N=\frac{\sum c}{(n1+0.1n2)d}$$

where

∑c is the total number of colonies counted on all selected plates;

n_1_ is the number of plates from the first selected dilution;

n_2_ is the number of plates from the second selected dilution;

d is the dilution factor corresponding to the first selected dilution;

0.1 is the weighting factor accounting for the tenfold difference between consecutive dilutions.

The method was performed in accordance with ISO 4833-1 standards [23].

### 2.9. Determination of Antimicrobial Activity of Lactic Acid Bacteria Isolates

The antimicrobial activity of the tested samples was evaluated using the disk-diffusion method. *Staphylococcus aureus* ATCC 25923, *Escherichia coli* ATCC 25922 and *Pseudomonas aeruginosa* ATCC 27853 were used as indicator microorganisms. Fresh (24 h) cultures of the test strains were standardized to 0.5 McFarland turbidity and evenly inoculated onto the surface of Mueller-Hinton agar (MHA). Sterile paper disks (6 mm diameter) were placed on the seeded agar and 20 µL of each test sample was applied onto each disk. Plates were pre-incubated at 4 °C for 2–4 h to allow diffusion of antimicrobial metabolites, and then incubated at 35 ± 2 °C for 18–24 h. Antimicrobial activity was assessed by the presence and size of the inhibition zone around the disks. All determinations were performed in three biological replicates.

Three sample variants were tested: native yoghurt, sterile-filtered yoghurt whey, and sterile-filtered whey neutralized to pH 7.0. To obtain sterile-filtered whey, the liquid phase of the yoghurt was separated and passed through a 0.22 µm membrane filter. To evaluate the contribution of acid-dependent metabolites, a portion of the sterile-filtered whey was adjusted to pH 7.0 and then used in the disk-diffusion assay. Comparing the activity of native yoghurt, sterile-filtered whey, and neutralized sterile-filtered whey allowed assessment of the contributions of the cellular fraction, soluble antimicrobial metabolites, and the acidic factor to the observed inhibitory effect.

Inhibition zone sizes were recorded in millimetres. For comparison, the inhibition radius was also calculated using the formula (D − 6)/2, where D is the total diameter of the inhibition zone in mm and 6 mm is the diameter of the paper disk. If no clearly defined inhibition zone was observed, antimicrobial activity was recorded as not detected. The methodological approach was based on a modified disk-diffusion test commonly used to evaluate the antagonistic activity of cell-free supernatants of lactic acid bacteria [24,25].

### 2.10. Statistical Analysis

Statistical analysis was performed using one-way analysis of variance (ANOVA) to evaluate the effect of formulation (YC, YM, YP, YE) within each storage day. When a significant effect was detected (*p* < 0.05), means were separated using Tukey’s honestly significant difference (HSD) post hoc test. Results are presented as mean ± standard deviation, and different superscript letters within the same column indicate statistically significant differences (*p* < 0.05).

All yoghurt formulations (YC, YM, YP, YE) were produced in three independent batches on different production days. Microbiological and antimicrobial assays were performed at the beginning of refrigerated storage (day 1) and after 14 days of storage. For each sampling point and each formulation, analyses were carried out in triplicate on samples taken from each batch, and the mean values were used for subsequent statistical treatment.

Data for water activity, water-holding capacity, syneresis were analyzed by two-way analysis of variance (ANOVA), with yoghurt formulation and storage day as fixed factors. When significant effects were detected (*p* < 0.05), mean comparisons were performed using Tukey’s post hoc test. All analyses were carried out using PRISMA 4.0.

## 3. Results

### 3.1. Nutritional Profile

The basic nutritional composition of the experimental yoghurts is presented in Table 2.

Fat content differed significantly between formulations (*p* < 0.001), with the control sample (YC) showing the highest fat level (5.3%) compared with the buttermilk- and extract-enriched variants (3.22–3.32%), which did not differ from each other. Dry matter also varied among samples (*p* = 0.046): YE had the highest DM, whereas YP showed the lowest value, while YC and YM occupied intermediate positions. In contrast, protein content did not differ significantly between treatments (*p* = 0.314), although a slight, non-significant increase was observed in the samples containing buttermilk concentrate and herbal extract, suggesting that the applied fortification mainly affected the distribution of fat and total solids rather than the overall protein level.

### 3.2. Determination of Active and Titratable Acidity

**Figure 2:**
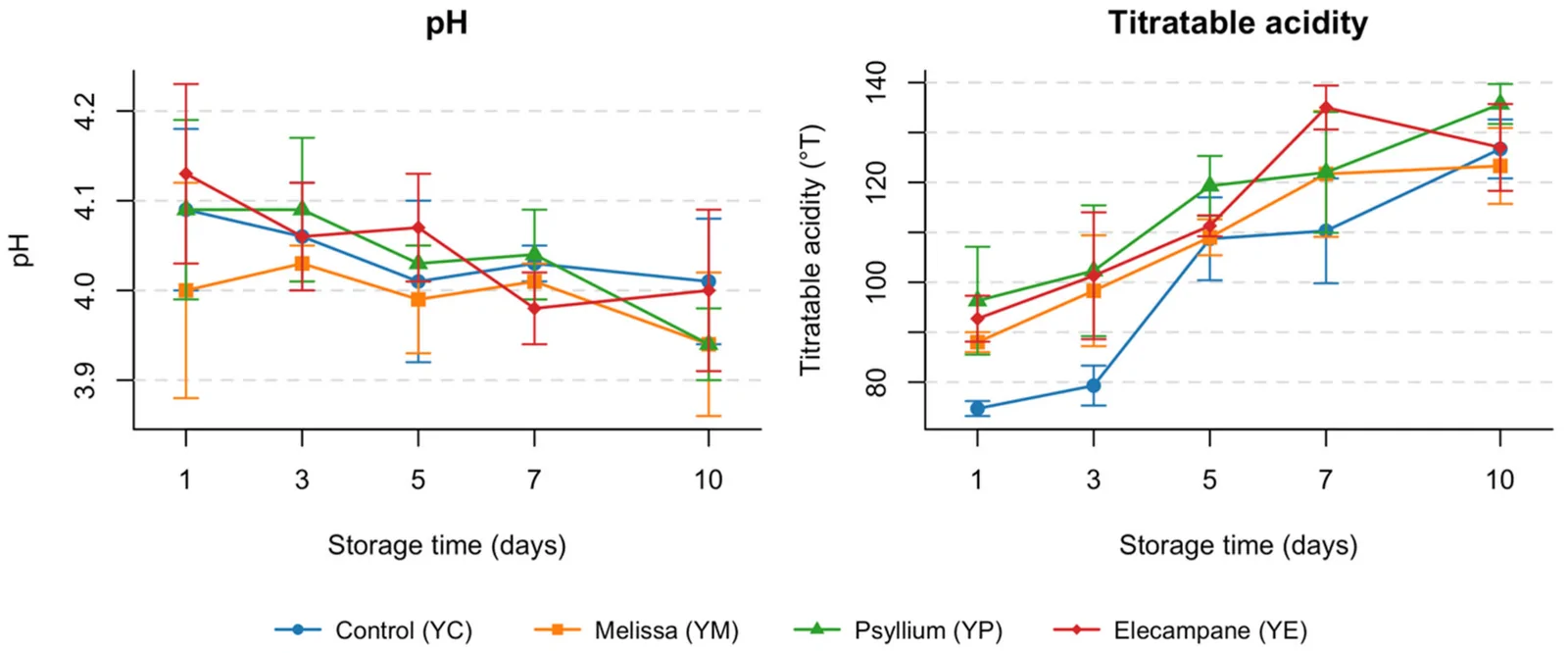
Active and titratable acidity of yoghurt samples.

The evolution of active and titratable acidity of the yoghurt samples during refrigerated storage is presented is shown in Figure 2.

Throughout the 10-day storage period, the pH values of all samples remained within a relatively narrow range, approximately from 3.9 to 4.2, indicating a typical level of acidification for fermented milk products. In the control yoghurt (YC), pH values were close to 4.1 on day 1, showed only minor fluctuations during storage and remained around 4.0 at day 10, indicating a stable acidification profile without signs of over-acidification. A similarly stable behaviour was observed in the yoghurts fortified with plant additives, with only slight formulation-dependent differences in absolute pH values.

Among the supplemented samples, the melissa (YM) and plantain (YP) yoghurts generally exhibited pH values comparable to or marginally lower than those of the control at later storage times, whereas the elecampane yoghurt (YE) tended to have pH values close to 4.0 throughout the experiment. These small differences suggest that the incorporation of plant extracts and buttermilk concentrate did not markedly alter the overall acid–base balance of the yoghurt matrix, although the slightly lower pH values in some fortified formulations may indicate a modest enhancement of acid development or buffering interactions between milk components and plant-derived substances. Despite these variations, no abrupt pH shifts were observed in any sample, and all products remained within the pH range typically reported for acceptable-quality yoghurt during refrigerated storage.

Titratable acidity increased in all formulations as storage progressed, confirming the continued metabolic activity of lactic acid bacteria after fermentation. In the control yoghurt, titratable acidity rose from approximately 75 °T on day 1 to about 127 °T by day 10, reflecting normal post-acidification during cold storage. A similar pattern was found in the fortified yoghurts, with YM, YP and YE reaching final values of roughly 123–136 °T, and YP and YE generally exhibiting the highest titratable acidity at the end of storage.

Taken together, the pH and titratable acidity data indicate that the incorporation of buttermilk concentrate and herbal extracts did not inhibit acid development in the yoghurts and, in the case of the plantain—and elecampane-enriched formulations, was associated with a slightly more pronounced increase in titratable acidity during storage. These observations suggest that the selected plant ingredients may provide a favorable environment for acid-producing microorganisms or subtly modify the buffering capacity of the yoghurt matrix without causing excessive acidification or destabilizing pH.

### 3.3. Determination of Water-Holding Capacity (WHC) and Syneresis

Water-holding capacity (WHC) of the yoghurt samples is summarized in Table 3.

At day 1, the control yoghurt (YC) exhibited significantly higher WHC compared with the yoghurts containing melissa, plantain and elecampane (YM, YP, YE), which formed a homogeneous group with lower values (*p* < 0.05). By day 3, WHC of the supplemented yoghurts (YM, YP, YE) increased and became significantly higher than that of the control, whereas no differences were detected among the three fortified formulations. On day 5, no statistically significant differences in WHC were observed between samples, indicating a similar water-retention behavior at this stage of storage. In contrast, at day 7, YC and YP showed the highest WHC and belonged to the same statistical group, YM had intermediate values, and YE demonstrated the lowest WHC, with all three groups differing significantly from each other. At the end of storage (day 10), WHC values tended to be higher overall, particularly in YP and YE, but the differences among formulations were not statistically significant, suggesting that prolonged storage attenuated the effect of plant additives on WHC.

A two-way ANOVA confirmed that both sample type and storage time significantly affected the WHC of yoghurt (*p* = 0.0058 and *p* < 0.001, respectively), and that the time-dependent changes in WHC differed between formulations, as indicated by a significant sample × time interaction (*p* < 0.001).

Syneresis of the yoghurt samples is summarized in Table 4.

Across all formulations, syneresis increased with storage time, from approximately 21–28% on day 1 to about 38–44% by day 10. At day 5, YM exhibited a slightly lower whey separation than YP (*p* < 0.05), while the other samples did not differ significantly. On day 7, YC and YE showed the highest syneresis, YP had intermediate values, and YM demonstrated the lowest whey separation (*p* < 0.05). By the end of storage (day 10), YE maintained significantly lower syneresis than the other formulations, whereas YC, YM and YP formed a homogeneous group with higher whey separation.

According to the two-way ANOVA, storage time had a strong effect on syneresis (*p* < 0.001), while the overall main effect of formulation was not significant when averaged across all sampling points (*p* = 0.42). However, the significant sample × time interaction (*p* < 0.001) indicates that the time-dependent increase in syneresis differed between formulations, supporting the interpretation that plant extracts modulated the evolution of whey separation during storage rather than its absolute level at any single time point.

### 3.4. Determination of Water Activity (a_w_)

Active water (a_w_) values of the yoghurt samples during refrigerated storage are presented in Table 5. For all formulations, a_w remained very high and close to unity, indicating that the products possessed a moisture level typical of fermented dairy foods. In the control yoghurt (YC), water activity fluctuated only slightly between approximately 0.973 and 0.991 over the 10-day storage period, with no consistent upward or downward trend. This pattern suggests that storage did not induce substantial changes in the binding of water in the control formulation.

The yoghurts containing plant additives likewise exhibited only small variations in *a_w_* during storage. In the melissa yoghurt (YM), water activity showed a slight dip on day 3, rose modestly on days 5–7, and then decreased again by day 10. The plantain yoghurt (YP) maintained *a_w_* values very similar to the control, with only minor day-to-day fluctuations, whereas the elecampane yoghurt (YE) tended to display slightly lower *a_w_* at the beginning of storage and values comparable to the other formulations at later time points. Overall, all samples remained within a very narrow *a_w_* interval of about 0.993–0.999, which is consistent with published data for yoghurt and indicates that the addition of plant ingredients did not meaningfully alter the water activity profile or the microbiological safety conditions of the products under refrigerated storage.

### 3.5. Determination of Colony-Forming Units (CFU)

The content of viable lactic acid microorganisms in the tested yoghurt samples at the beginning of storage ranged from 1.10 ± 0.06 × 10^8^ to 4.10 ± 0.21 × 10^8^ CFU/mL. Yoghurts with a combined base (milk + buttermilk concentrate) showed higher CFU counts than milk-only formulations, likely due to the nutrient enrichment from buttermilk concentrate. After 14 days of storage, a decrease in this parameter was observed in all samples except the one containing *Plantago major* L.

In the control sample, the number of lactic acid bacteria decreased from 2.32 ± 0.12 × 10^8^ to 1.72 ± 0.09 × 10^8^ CFU/mL, corresponding to a 25.9% reduction (*p* = 0.0027). In the sample with *Melissa officinalis* L., the viable cell count decreased from 4.10 ± 0.21 × 10^8^ to 2.85 ± 0.14 × 10^8^ CFU/mL, i.e., by 30.5% (*p* = 0.0016). The most pronounced reduction in lactic acid microflora was recorded in the *Inula helenium* L. variant, where CFU declined from 3.30 ± 0.17 × 10^8^ to 1.07 ± 0.05 × 10^8^ CFU/mL, or by 67.6% relative to the initial level (*p* = 0.0007).

In contrast to the aforementioned samples, the yoghurt containing *Plantago major* L. exhibited the opposite trend: by day 14 of storage, the content of viable lactic acid microorganisms increased from 1.10 ± 0.06 × 10^8^ to 4.56 ± 0.23 × 10^8^ CFU/mL, corresponding to a 314.5% increase (*p* = 0.0008).

**Figure 3:**
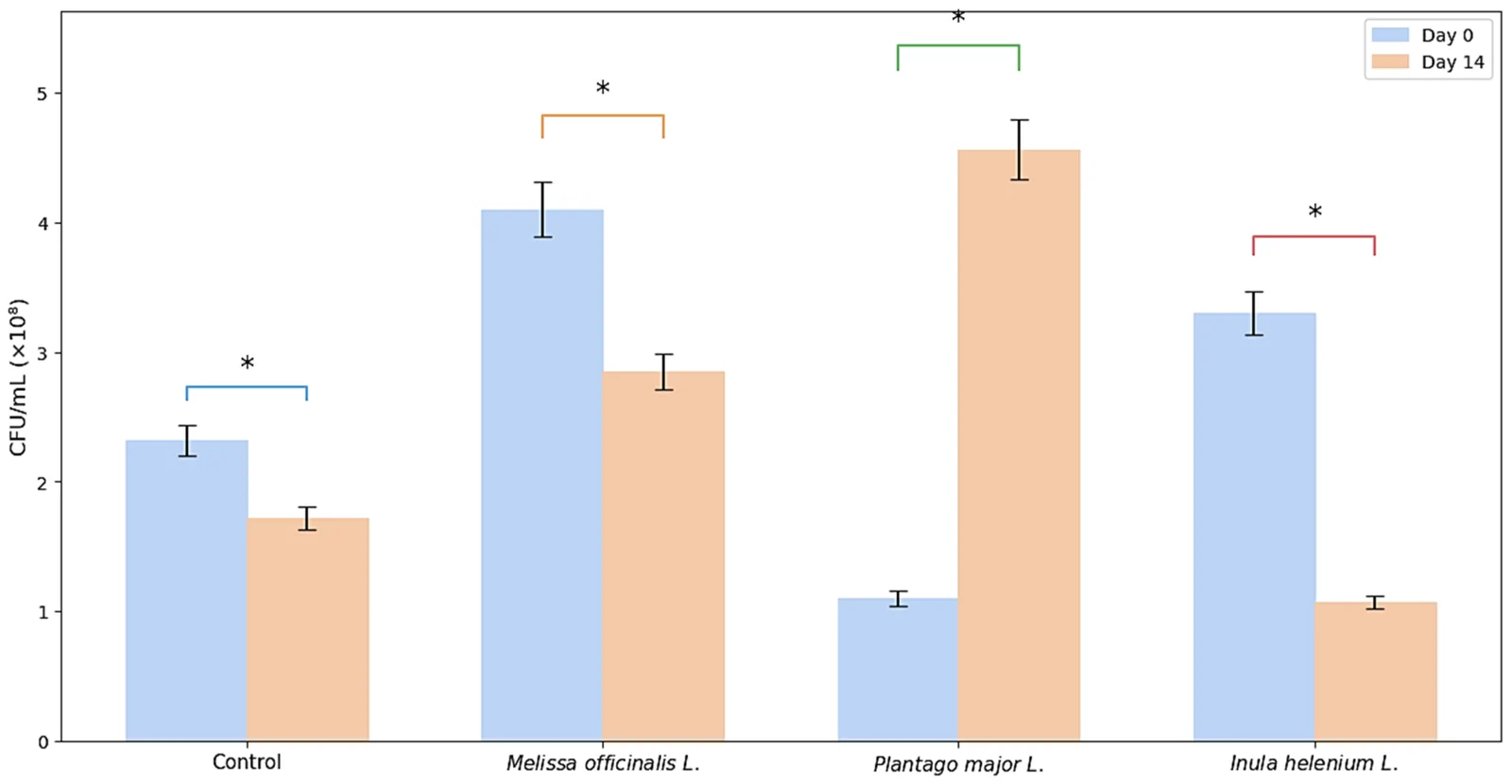
Changes in the content of viable lactic acid microorganisms in yoghurt samples at the beginning of storage and after 14 days. * indicates statistically significant differences (*p* < 0.05) according to one-way ANOVA followed by Tukey’s post hoc test.

These results indicate divergent effects of plant components on the stability of lactic acid microflora during storage. While the control sample, as well as the variants with *Melissa officinalis* L. and *Inula helenium* L., showed a statistically significant decrease in viable cell counts, the addition of *Plantago major* L., conversely, was accompanied by a statistically significant increase in CFU (Figure 3).

Thus, among the tested plant additives, plantain (*Plantago major* L.) exerted the most favorable effect on the preservation of lactic acid bacteria, whereas elecampane (*Inula helenium* L.) demonstrated the most pronounced inhibitory effect on the viability of the starter microflora.

### 3.6. Determination of Antimicrobial Activity of Lactic Acid Bacteria Isolates

Based on the disk-diffusion assay, the tested samples exhibited selective antimicrobial activity, most pronounced against *Staphylococcus aureus*. Clear inhibition zones against *S. aureus* were recorded for all native yoghurt variants. The maximum inhibition radius was observed in the sample with *Plantago major* L., measuring 5.8 ± 0.5 mm, whereas the control sample showed a radius of 5.3 ± 0.5 mm. Slightly lower activity was found in yoghurts with *Inula helenium* L. and *Melissa officinalis* L., with inhibition radii of 4.8 ± 0.5 mm and 4.3 ± 0.5 mm, respectively. Thus, based on the extent of anti-staphylococcal activity, the native yoghurt samples can be ranked as follows: *Plantago major* L. > control > *Inula helenium* L. > *Melissa officinalis* L.

**Figure 4:**
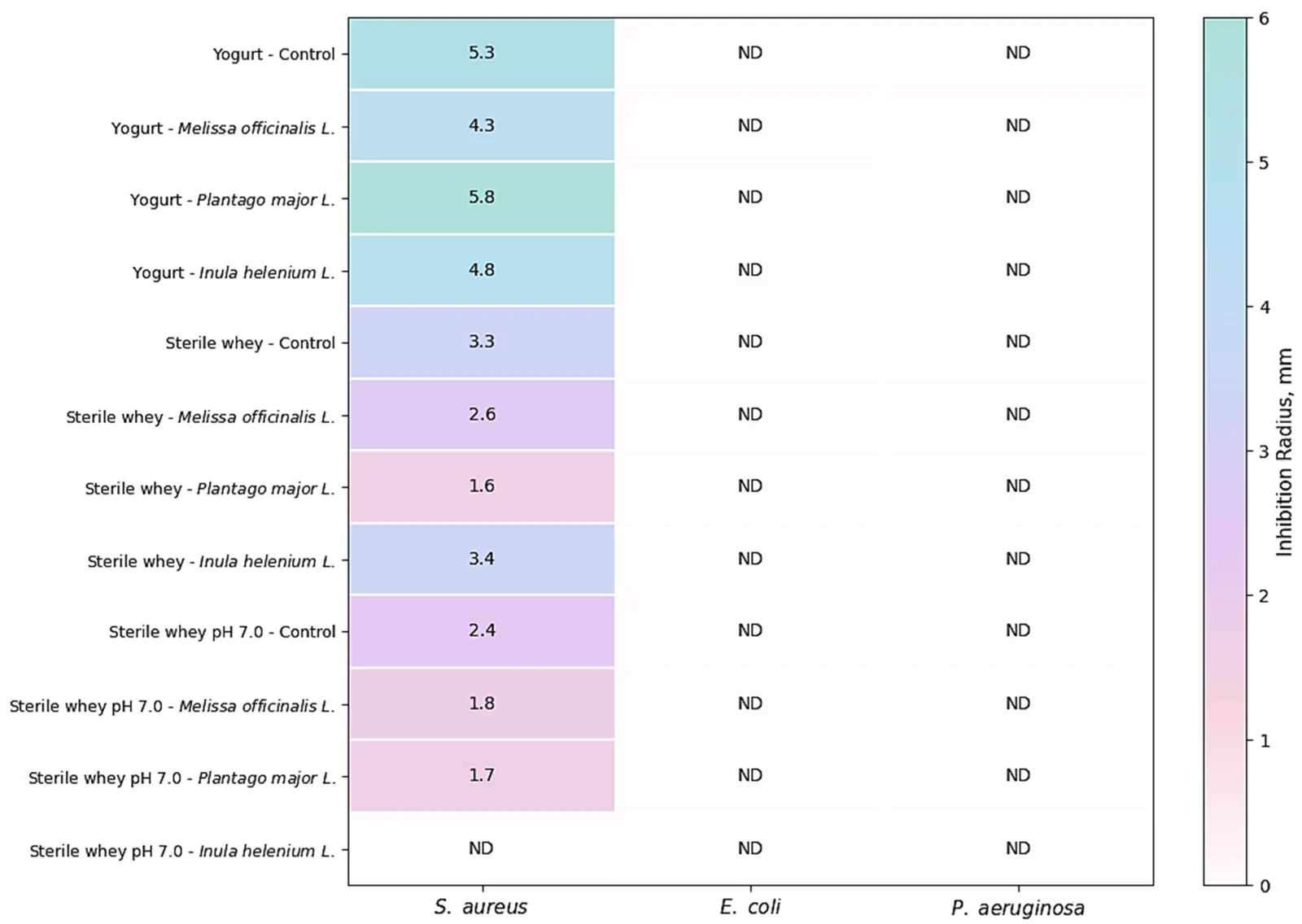
Heat map of antimicrobial activity of yoghurt samples against *Staphylococcus aureus*, *Escherichia coli*, and *Pseudomonas aeruginosa* based on inhibition radius (mm).

After sterile filtration of the wheys, the antimicrobial effect against *S. aureus* was retained but reduced in magnitude. For the sterile whey of the control yoghurt, the inhibition radius was 3.3 ± 0.5 mm; for the sample with *Inula helenium* L., 3.4 ± 0.4 mm; for the sample with *Melissa officinalis* L., 2.6 ± 0.4 mm; and the minimum value was observed for the whey of the yoghurt with *Plantago major* L. (1.6 ± 0.5 mm). Thus, removal of the cellular fraction and transition to sterile whey were accompanied by a decrease in antimicrobial activity compared to the original yoghurt, although the anti-staphylococcal effect was preserved in all tested variants (Figure 4).

Additional neutralization of the sterile whey to pH 7.0 led to a further decrease in antimicrobial activity. For the control sample, the inhibition radius against *S. aureus* decreased to 2.4 ± 0.4 mm; for the sample with *Melissa officinalis* L., to 1.8 ± 0.4 mm; and for the variant with *Plantago major* L., to 1.7 ± 0.4 mm. For the neutralized whey of the yoghurt with *Inula helenium* L., a statistically significant inhibition zone was not detected. This reduction in activity after adjusting pH to neutrality indicates a substantial contribution of acid-dependent metabolites to the antimicrobial effect of the tested samples. At the same time, the preservation of weak residual activity in some variants suggests an additional contribution of non-acidic antimicrobial factors.

Against *Escherichia coli* and *Pseudomonas aeruginosa*, no statistically significant antimicrobial activity was detected in any of the tested variants, including native yoghurt, sterile whey, and neutralized sterile whey. This indicates that the antagonistic action of the investigated fermented dairy systems is predominantly targeted and expressed mainly against the Gram-positive test strain *S. aureus*. Overall, the results show that the highest antimicrobial potential is characteristic of native yoghurts, whereas sterile filtration and especially pH neutralization are accompanied by a progressive weakening of the inhibitory effect.

## 4. Disscusion

### 4.1. Nutritional Profile

Fats, dry matter and protein contents of the developed yoghurts follow trends that are consistent with previous reports on buttermilk-fortified and composition-modified yoghurts. The significantly higher fat content in the control sample and the uniformly lower fat levels in all fortified variants are expected, since partial substitution of whole milk by buttermilk concentrate and protein-rich ingredients typically reduces fat while maintaining acceptable structure and mouthfeel. Similar fat reductions without loss of quality have been reported in yoghurts enriched with whey proteins or buttermilk powder [1,2].

Numerical differences in dry matter among formulations, with YE tending to have the highest DM and YP the lowest, are in line with the well-known role of total solids and added functional ingredients in modulating gel strength and water retention, even though these differences were not statistically significant at the formulation level. Studies on yoghurts fortified with whey protein concentrates or buttermilk powder demonstrate that higher solids are associated with improved rheological properties and water-holding capacity, which supports the interpretation that the slightly elevated DM in YE may contribute to its comparatively favorable physical stability observed during storage. The lack of significant variation in protein content, despite numerically higher values in the fortified samples, agrees with reports that many modern yoghurt formulations differ more in microstructure and texture than in bulk protein percentage once a relatively high baseline level is established. In this context, fortification with buttermilk concentrate and plant extracts in our study appears to adjust the balance of fat and total solids while preserving a consistently high protein level across all formulations, in accordance with current strategies for designing functional yoghurts [26].

Carbohydrate and ash contents showed no significant formulation effect for carbohydrates and only a modest but significant increase in ash in the YE sample compared with the control, which is consistent with the additional mineral contribution from buttermilk and herbal solids. The numerically higher carbohydrate levels in the fortified samples can be attributed to residual lactose from buttermilk and to plant-derived carbohydrates, but these differences were not large enough to translate into significant changes in calculated energy density, which remained within a narrow range for all products. Together, these observations indicate that the incorporation of buttermilk concentrate and herbal extracts primarily redistributes fat and solids within the yoghurt matrix without markedly altering protein content or overall caloric value, while providing a compositional basis for the textural and stability effects discussed in subsequent sections [27].

### 4.2. Active and Titratable Acidity

Changes in pH and titratable acidity observed in our yoghurts were generally consistent with previous reports on dairy and plant-enriched yoghurts. In all formulations, pH remained within the typical range of 3.8–4.5 during cold storage, while titratable acidity gradually increased, reflecting the ongoing metabolic activity of lactic acid bacteria after fermentation. This behaviour agrees with findings for yoghurts supplemented with various plant ingredients, where storage is usually accompanied by a slow decrease in pH and a concomitant rise in titratable acidity due to continued lactic acid production [28].

The more pronounced acidification in plantain- and elecampane-fortified samples, compared with the control yoghurt, is in line with several studies showing that fibre- and herb-enriched yoghurts tend to exhibit lower pH and higher titratable acidity than plain formulations. Plantain husk has been reported to enhance lactic acid formation and reduce pH in both dairy and plant-based yoghurts, which is attributed to the presence of highly branched arabinoxylans that support the survival and metabolic activity of *S. thermophilus* and *Lactobacillus delbrueckii* ssp. *bulgaricus*. Similarly, yoghurts supplemented with different herbal extracts were shown to acidify faster and to reach higher titratable acidity than control samples, indicating that plant bioactives can stimulate starter culture activity or modify the buffering capacity of the matrix [29].

At the same time, the absence of drastic pH drops in our study suggests that the level of plant supplementation did not lead to over-acidification, which is important for maintaining acceptable sensory quality during storage. This is comparable to the observations of Devnani et al. and other authors, who reported that cow-milk yoghurts fortified with fibres or plant ingredients usually remain within an acceptable pH window despite increased titratable acidity, owing to the buffering effect of milk proteins and minerals. Taken together, these comparisons indicate that melissa, plantain and elecampane can be incorporated into yoghurt without impairing acidification kinetics; on the contrary, plantain and elecampane appear to promote a slightly more intense but still technologically desirable acid development during storage [30].

### 4.3. Water-Holding Capacity (WHC) and Syneresis

In the present study, the incorporation of plant ingredients had a dynamic but generally favourable effect on the water-holding capacity of yoghurt during storage. Initially, the control sample exhibited the highest WHC, whereas melissa-, plantain- and elecampane-enriched yoghurts showed significantly lower values, which may reflect the time required for plant fibres and bioactive compounds to become fully integrated into the protein gel network. As storage progressed, WHC of the supplemented formulations increased and, from day 3 onward, was comparable to or higher than that of the control, especially in the plantain and elecampane yoghurts at later time points. This pattern is consistent with previous reports indicating that soluble fibres and herbal components can strengthen the casein network and improve water retention in fermented milk products by promoting a more compact gel structure and enhancing serum phase immobilization [31].

The two-way ANOVA for WHC showed that both sample type and storage time had significant main effects, and, importantly, that there was a significant interaction between these factors. This indicates that the development of WHC during storage depended on the formulation, i.e., the plant extracts and buttermilk concentrate modified the time-dependent changes in water retention. In general, all yoghurts showed an initial decrease or fluctuation in WHC followed by partial recovery at later storage days, in line with previous reports on the dynamic restructuring of yoghurt gels during cold storage. The buttermilk- and extract-enriched formulations tended to reach WHC values equal to or higher than the control at the end of storage, suggesting that the combined presence of milk proteins, buttermilk components and plant-derived polysaccharides or fibres can strengthen the gel network and enhance serum immobilization over time. In particular, the plantain- and elecampane-enriched yoghurts exhibited higher WHC values than the control on day 10, which is consistent with the ability of soluble fibers and inulin-type fructans to interact with casein micelles, increase total solids and promote a more cohesive microstructure with reduced free serum phase.

The evolution of syneresis over time followed the opposite trend to WHC, with all samples showing a gradual increase in whey separation during storage, as typically reported for set-type yoghurts. Nevertheless, the fortified yoghurts, particularly those containing melissa and elecampane, tended to exhibit lower or comparable syneresis than the control at specific time points, despite the overall rise in serum separation with time. Similar reductions in syneresis have been described for yoghurts enriched with plant fibres, plantain husk and various herbal extracts, where additional total solids and hydrocolloids increase the viscosity of the continuous phase and limit whey expulsion. In our study, the comparatively low syneresis of the elecampane sample at the end of storage suggests that this ingredient may contribute to a more stable gel network, which aligns with the concept that certain plant-derived polysaccharides and phenolic compounds can interact with milk proteins and improve the physical stability of yoghurt gels [1,32].

For syneresis, storage time exerted a dominant effect, whereas the overall main effect of formulation averaged across all sampling points was not significant. Nevertheless, the significant sample × time interaction indicates that the rate and extent of whey separation during storage differed between formulations. All yoghurts showed a progressive increase in syneresis, reflecting gradual rearrangements and shrinkage of the protein network under cold storage. At the final sampling point, the elecampane-enriched yoghurt showed markedly lower syneresis than the other formulations, suggesting that this extract may contribute to improved gel stability, possibly through additional solids, fibre-like components or specific interactions with milk proteins that limit serum expulsion.

Taken together, the WHC and syneresis data indicate that the addition of melissa, plantain and elecampane did not compromise the physical stability of the yoghurts and in some cases conferred a technological advantage. The increase in WHC and the relatively lower syneresis observed in the plantain- and elecampane-containing samples towards the end of storage support the potential of these ingredients as functional texturizing agents capable of improving water retention and reducing whey separation without the need for synthetic stabilizers [32]. Overall, the extent of syneresis observed in the present study (approximately 20–44%) was lower or comparable to the values reported for fortified yoghurts in previous studies, where whey separation may reach 35–50% or even higher depending on formulation and storage conditions. This reduction in whey separation can be attributed, at least in part, to the incorporation of buttermilk concentrate, whose high protein content and milk fat globule membrane components are known to strengthen the gel network and improve water retention in yoghurt matrices.

### 4.4. Water Activity in Samples

Water activity values measured in the present yoghurts are in good agreement with those reported for fresh fermented milk products. Across all formulations and storage times, *a_w_* remained within a very narrow interval close to 0.99, which fits well within the commonly cited range of approximately 0.97–0.99 for plain yoghurt. Comparable *a_w_* levels (around 0.980–0.991) have also been described for both dairy and plant-based yoghurts, indicating that a high availability of water is generally maintained regardless of the underlying matrix and that the values obtained here are typical for spoonable refrigerated yoghurts [26]. Although two-way ANOVA indicated a significant effect of formulation on a_w_ (F_3,123,12_ = 5.73, *p* = 0.011), while storage time had no significant influence (*p* > 0.05), the absolute differences among samples were small and did not shift the products out of the high-moisture range.

Unlike formulations in which water activity is deliberately reduced, the products in this study clearly correspond to high-moisture yoghurts. Patents and technical documents on “reduced water activity yoghurt” describe systems with a_w_ values below roughly 0.85, achieved by substantially increasing total solids and incorporating humectants to obtain shelf-stable products in which the growth of pathogenic microorganisms is suppressed. By contrast, the a_w_ values of about 0.99 observed here are typical of fresh yoghurts designed for refrigerated distribution rather than ambient storage, and fall within the general range reported for high-moisture foods. From a sensory perspective, preserving such high water activity is advantageous, because pronounced reductions in a_w_ in yoghurt matrices have been associated with excessively firm, dense structures and mouthfeel changes resembling those of partially dehydrated products [33,34,35].

### 4.5. Determination of Colony-Forming Units (CFU)

The viability of lactic acid bacteria (LAB) is a critical quality indicator for yoghurt, directly influencing its probiotic functionality and shelf-life. In our study, initial LAB counts ranged from 1.10 ± 0.06 × 10^8^ to 4.10 ± 0.21 × 10^8^ CFU/mL, meeting probiotic standards and confirming successful fermentation. Notably, yoghurts formulated with a combined base (milk + buttermilk concentrate) exhibited higher initial viability than milk-only controls. This enhancement is attributed to buttermilk concentrate, which enriches the matrix with growth factors, milk fat globule membrane components, and bioactive peptides that support LAB proliferation [8,17,19].

Storage dynamics revealed divergent effects of plant additives. While control and *Melissa officinalis* L./*Inula helenium* L. samples showed significant viability declines (up to 67.6% in *Inula*), *Plantago major* L. uniquely promoted a 314.5% CFU increase [15,28]. The decline in control/*Inula* aligns with cold storage stress (acid accumulation, nutrient depletion), while *Inula*’s sharp reduction may reflect inhibitory sesquiterpene lactones [28,30]. Conversely, *Plantago*’s proliferation suggests a prebiotic-like effect: its polysaccharides (mucilages) and phenolics likely improved water-holding capacity, shielding LAB from acid/oxidative damage. This contrasts with extracts that reduce viability due to antimicrobial constituents, highlighting *Plantago* as superior for functional yoghurt [16,31,32].

The markedly higher LAB counts in the yoghurt with *Plantago major* L. extract may be related to the presence of plant-derived polysaccharides and phenolic compounds, as Plantago species are known to be rich in such components and similar molecules have been reported to exert prebiotic-like effects on lactic acid bacteria in other systems. However, this mechanism was not directly evaluated in the present study and should be considered a hypothesis to be addressed in future work [30,32].

Antimicrobial activity was selective against Gram-positive *S.aureus* but absent against Gram-negative *E. coli*/*P. aeruginosa*, consistent with LAB antagonism where Gram-negative outer membranes block metabolite penetration [25,27]. Highest activity was in *Plantago* native yoghurt, correlating with higher LAB populations and metabolite accumulation. Sterile filtration reduced activity but retained inhibition, confirming contributions from both cellular components and soluble metabolites. pH neutralization drastically reduced activity (with *Inula* losing it entirely), proving acid-dependent metabolites dominate, while residual activity in *Plantago* implies stable non-acidic factors (bacteriocins, phenolics) [11,12].

The synergistic effect of buttermilk concentrate and *Plantago major* L. emerged as optimal: the concentrate provided a nutrient-rich base elevating baseline viability, while *Plantago* further enhanced stability and antimicrobial potency via exopolysaccharides and phenolics. In contrast, *Inula* compromised viability despite bioactivity, indicating a trade-off between extract potency and starter compatibility.

### 4.6. Determination of Antimicrobial Activity of Lactic Acid Bacteria Isolates

The tested yoghurts showed selective antimicrobial activity, strongly inhibiting Gram-positive *Staphylococcus aureus* but not Gram-negative *E. coli* or *P. aeruginosa* [25,27]. The highest activity was in the *Plantago major* L. variant (5.8 ± 0.5 mm), followed by control, *Inula helenium* L., and *Melissa officinalis* L. [11,13]. This selectivity reflects the typical susceptibility of Gram-positive bacteria to LAB metabolites, while Gram-negative outer membranes block penetration.

Sterile filtration reduced activity but retained inhibitory effect, confirming contribution of both cellular components and soluble metabolites [25]. Neutralization to pH 7.0 further drastically reduced activity, with *Inula* losing it entirely, proving that acid-dependent metabolites (lactic/acetic acids) are the main antimicrobial drivers [24,25]. Residual activity in *Plantago* and control after neutralization suggests additional non-acidic factors like bacteriocins or plant phenolics [11,12].

### 4.7. Limitations of the Study

This study has several limitations that should be acknowledged. First, the experimental design did not include a separate yoghurt formulation without buttermilk concentrate, which would allow a full factorial separation of the effects of the concentrated milk–buttermilk base and the herbal extracts. Second, the scope of the funded project underlying this work was focused on physicochemical, lactic acid bacteria viability and antimicrobial parameters; therefore, antioxidant assays and quantitative determination of dietary fibre fractions (SDF and IDF) were not performed, and the mechanistic role of plant-derived components in fibre-related functionality remains to be clarified in future studies. Third, fermentation was monitored mainly at the end of cold storage, and dynamic measurements of pH, water activity and colour during fermentation were not included; according to the project calendar, these time-resolved analyses are planned for the next phase of our research and will be reported elsewhere. Taken together, these limitations should be considered when interpreting the findings and indicate several directions for further work on buttermilk- and herb-fortified yoghurt systems.

Another limitation of this work is that storage stability was assessed only for 14 days, so it remains to be confirmed whether the observed trends persist over the longer storage times (21–30 days) typical of commercial yoghurt.

## 5. Conclusions

Yoghurts fortified with *Melissa officinalis* L., *Plantago major* L. (plantain), and *Inula helenium* L. (elecampane) demonstrated physicochemical and microbiological characteristics compatible with high-quality fermented milk products over 10 days of refrigerated storage. The addition of plant ingredients did not inhibit acid development; on the contrary, *Plantago* and *Inula* slightly enhanced post-acidification while maintaining pH within the acceptable range for yoghurt. Water-holding capacity and syneresis data indicated that fortified samples, especially those with *Plantago* and *Inula*, achieved equal or better physical stability than the control, suggesting their potential contribution to texture and whey retention.

Microbiologically, the combined base (milk + buttermilk concentrate) provided a nutrient-rich matrix that enhanced initial LAB viability. Among the plant additives, *Plantago major* L. was the most favorable under the conditions tested, showing a 314% higher viable LAB count during storage and maintaining anti-staphylococcal activity. In contrast, *Inula helenium* L., despite its bioactive profile, reduced starter culture viability, indicating a possible trade-off between antimicrobial action and compatibility with starter culture. Antimicrobial testing revealed selective activity against *Staphylococcus aureus* in fortified samples, whereas no inhibition of *E. coli* or *Pseudomonas aeruginosa* was detected, indicating a limited antimicrobial spectrum under the tested conditions.

In conclusion, the combination of *Plantago major* L. extract with a buttermilk-enriched milk base appears particularly promising for the development of yoghurt with enhanced LAB viability and measurable antimicrobial activity against *S. aureus* under the tested conditions. *Melissa* and low doses of *Inula* can also be incorporated to improve physical stability, provided their effects on starter culture viability are carefully monitored. Overall, these findings highlight the importance of selecting plant–dairy combinations that are compatible with lactic acid bacteria in order to obtain yoghurt products with improved physicochemical properties, LAB viability and microbiological stability, without overstating broad bioprotective or safety claims. However, the small inhibition zones and the absence of activity against *E. coli* and *P. aeruginosa* indicate that the antimicrobial effect is modest and spectrum-limited and should not be interpreted as broad bioprotection. Moreover, no sensory evaluation or extended shelf-life testing was performed, so the industrial relevance of these formulations should be confirmed in future studies.

## Figures and Tables

**Table 1:** Chemical composition of raw materials, %.

	Parameters	Skimmed Cow’s Milk	Goat’s Milk	Buttermilk Concentrate
1	TS	11.66 ± 0.02	5.95 ± 0.03	10.16 ± 0.01
2	Protein	3.98 ± 0.03	1.36 ± 0.01	2.89 ± 0.01
3	Fat	0	2.18 ± 0.01	0.69 ± 0.01

**Table 2:** Nutritional profile of samples, %.

	YC	YM	YP	YE
Fat	5.3 ± 0.41 ^b^	3.32 ± 0.40 ^a^	3.27 ± 0.36 ^a^	3.22 ± 0.39 ^a^
DM	15.86 ± 0.90 ^a^	15.99 ± 1.05 ^a^	14.96 ± 0.73 ^a^	16.89 ± 1.21 ^a^
Protein	5.99 ± 0.83 ^a^	6.83 ± 0.85 ^a^	6.13 ± 0.55 ^a^	7.36 ± 1.17 ^a^
Carbohydrate	3.92 ± 0.29 ^a^	5.12 ± 0.41 ^a^	4.86 ± 0.98 ^a^	5.56 ± 0.73 ^a^
Ash	0.65 ± 0.04 ^a^	0.72 ± 0.01 ^a^	0.7 ± 0.01 ^a^	0.75 ± 0.02 ^b^
Energy, kcal/100 g	87.3 ± 7.2 ^a^	77.7 ± 7.5 ^a^	73.4 ± 5.5 ^a^	80.7 ± 9.1 ^a^

YC—yoghurt control sample; YM—yoghurt with melissa extract; YP—yoghurt with plantain extract; YE—yoghurt with elecampane. One-way ANOVA: Fat—*p* < 0.001; DM—*p* = 0.046; Protein—*p* = 0.314 (NS); Carbohydrate—*p* = 0.412 (NS); Ash—*p* = 0.003; Energy—*p* = 0.287 (NS). Values are expressed as mean ± standard deviation (*n* = 3). Different superscript letters within the same row indicate statistically significant differences among samples according to one-way ANOVA followed by Tukey’s HSD post hoc test (*p* < 0.05). NS—not significant.

**Table 3:** WHC of the samples (%).

Sample	1st Day	3rd Day	5th Day	7th Day	10th Day
YC	51.86 ± 3.11 ^a^	44.73 ± 1.24 ^b^	40.18 ± 6.18 ^a^	48.00 ± 1.48 ^a^	53.57 ± 1.15 ^a^
YM	38.95 ± 1.62 ^b^	49.33 ± 4.72 ^a^	44.00 ± 1.15 ^a^	43.53 ± 0.67 ^b^	52.68 ± 8.16 ^a^
YP	38.19 ± 0.83 ^b^	52.98 ± 2.64 ^a^	44.10 ± 1.11 ^a^	46.37 ± 1.48 ^a^	60.31 ± 1.40 ^a^
YE	40.90 ± 0.19 ^b^	54.60 ± 3.14 ^a^	38.50 ± 2.13 ^a^	38.03 ± 0.35 ^c^	56.24 ± 1.49 ^a^

YC—yoghurt control sample; YM—yoghurt with melissa extract; YP—yoghurt with plantain extract; YE—yoghurt with elecampane. Values are expressed as mean ± standard deviation (*n* = 3; for some time points *n* = 6 as indicated in the text). Different superscript letters within the same column indicate significant differences among yoghurt samples according to one-way ANOVA followed by Tukey’s HSD test (*p* < 0.05). Two-way ANOVA (factors: sample type and storage time) showed significant main effects of sample (*p* = 0.0058) and storage time (*p* < 0.001), as well as a significant sample × time interaction (*p* < 0.001).

**Table 4:** Syneresis of the yoghurt.

Sample	1st Day	3rd Day	5th Day	7th Day	10th Day
YC	21.05 ± 4.07 ^a^	27.77 ± 1.88 ^a^	34.57 ± 0.85 ^a^	38.83 ± 0.59 ^a^	43.67 ± 1.46 ^a^
YM	28.30 ± 1.67 ^a^	27.06 ± 4.03 ^a^	34.00 ± 1.15 ^b^	33.52 ± 0.69 ^c^	44.00 ± 1.15 ^a^
YP	23.46 ± 2.11 ^a^	28.58 ± 1.32 ^a^	37.02 ± 1.13 ^a^	36.33 ± 1.50 ^b^	44.10 ± 1.11 ^a^
YE	23.71 ± 3.38 ^a^	28.90 ± 1.68 ^a^	34.33 ± 1.29 ^a^	38.03 ± 0.35 ^a^	38.50 ± 2.13 ^b^

YC—yoghurt control sample; YM—yoghurt with melissa extract; YP—yoghurt with plantain extract; YE—yoghurt with elecampane. Values are expressed as mean ± standard deviation (*n* = 3). Different superscript letters within the same column indicate significant differences among yoghurt samples according to one-way ANOVA followed by Tukey’s HSD test (*p* < 0.05) Two-way ANOVA (factors: sample type and storage time) revealed a highly significant effect of storage time on syneresis (*p* < 0.001) and a significant sample × time interaction (*p* < 0.001), whereas the main effect of sample type averaged across storage times was not significant (*p* = 0.42).

**Table 5:** Water activity (a_w_) results.

Sample	Day	a_w (Mean ± SD)
YC (control)	1	0.991 ± 0.0086
	3	0.986 ± 0.0016
	5	0.991 ± 0.0009
	7	0.982 ± 0.0013
	10	0.973 ± 0.0033
YM (melissa)	1	0.982 ± 0.0016
	3	0.980 ± 0.0017
	5	0.986 ± 0.0030
	7	0.984 ± 0.0008
	10	0.987 ± 0.0015
YP (plantain)	1	0.983 ± 0.0037
	3	0.970 ± 0.0009
	5	0.930 ± 0.0075
	7	0.935 ± 0.0024
	10	0.938 ± 0.0017
YE (elecampane)	1	0.954 ± 0.0022
	3	0.962 ± 0.0033
	5	0.970 ± 0.0008
	7	0.976 ± 0.0018
	10	0.976 ± 0.0064

Values are expressed as mean ± standard deviation (*n* = 2–3). No statistically significant differences in water activity were detected among yoghurt samples within the same storage day (*p* ≥ 0.05) Two-way ANOVA (factors: formulation and storage day) applied to mean water activity values showed a significant effect of yoghurt formulation on a_w_ (F_3,12_ = 5.73, *p* = 0.011), whereas storage time had no significant effect (F_4,12_ = 0.29, *p* = 0.878).

## Data Availability

The original contributions presented in the study are included in the article, further inquiries can be directed to the corresponding author.
